# The general-relativistic case for super-substantivalism

**DOI:** 10.1007/s11229-021-03398-9

**Published:** 2021-10-16

**Authors:** Patrick M. Duerr, Claudio Calosi

**Affiliations:** 1 Oriel College, University of Oxford, Oxford, UK; 2 Institute for Theoretical Philosophy, University of Bremen, Bremen, Germany; 3 Department of Philosophy, University of Geneva, Geneva, Switzerland

**Keywords:** Super-substantivalism, Ontological dependence, Fundamentality, Geometric approach to spacetime, General relativity

## Abstract

Super-substantivalism (of the type we’ll consider) roughly comprises two core tenets: (1) the physical properties which we attribute to matter (e.g. charge or mass) can be attributed to spacetime *directly*, with no need for matter as an extraneous carrier “on top of” spacetime; (2) spacetime is more fundamental than (ontologically prior to) matter. In the present paper, we revisit a recent argument in favour of super-substantivalism, based on General Relativity. A critique is offered that highlights the difference between (various accounts of) fundamentality and (various forms of) ontological dependence. This affords a metaphysically more perspicuous view of what super-substantivalism’s tenets *actually* assert, and how it may be defended. We tentatively propose a re-formulation of the original argument that not only seems to apply to all classical physics, but also chimes with a standard interpretation of spacetime theories in the philosophy of physics.

## Introduction

Super-substantivalism is a view (or rather: a family of views) about the relation between spacetime and material objects (such as neutrons, electromagnetic waves or axolotls). As a first pass, we can roughly distinguish three variants^1^:

**The Identity View**: Analogously to how water is identical to $H_{2} O$ molecules, material objects are *identical to* spacetime regions.

**The Constitution View**: Analogously to how a statue is constituted by a lump of matter, material objects are *constituted by* spacetime regions.^2^

**The Priority View**: Analogously to how facts about chemistry or biology are often viewed as less fundamental then, or derivative of physical facts (say, atoms and their interactions), material objects are *derivative of* spacetime regions. Equivalently, spacetime regions are prior to (or more fundamental than^3^) material objects.

These views clearly differ. We will return on their relations in Sect. 5.

Historically, the Identity View is arguably the most widely espoused form of super-substantivalism. Descartes, Spinoza and Alexander held versions of it; contemporary discussions and endorsements are found in Quine (1981, p. 17), Field (1984, p. 74), Lewis (1986, p. 76), Sider (2001, p. 110), Skow (2005), Schaffer (2009), and Nolan (2014, pp. 98–100). Gilmore (2014) explores the Constitution View at length. Lehmkuhl’s (2018) recent defence of the Priority View stands out. Unlike most other presentations of super-substantivalism, it doesn’t primarily draw on a priori considerations. Rather, according to Lehmkuhl, priority super-substantivalism is favoured by (one of) our best spacetime theories—Einstein’s theory of General Relativity (GR).

Recent progress in analytic metaphysics—especially with regards to ontological dependence and fundamentality—sheds new light on the arguments. On the one hand, *some* (in principle plausible) metaphysical stances towards dependence and fundamentality significantly strengthen (and generalise) Lehmkuhl’s main argument. *Other* stances (in their own right, in principle likewise plausible), on the other hand, vitiate it.

We intend the present paper as an explorative development—and friendly amendment—of Lehmkuhl's stance. He writes:

Different ways of cashing out ‘ontologically prior’ correspond to different ways of extending MESC [Minimal Extension of the Supersubstantivalist core commitment] further, to different concreter versions of supersubstantivalism (Lehmkuhl, 2018, p. 28).

Below, we’ll discuss different ways of cashing out the crucial notions of dependence—a notion that, as we’ll see, Lehmkuhl himself directly relates to priority—and priority. Special attention will be given to spelling out the most promising variants and extensions of priority super-substantivalism.

Besides an analysis and qualified defence of priority super-substantivalism, our paper can be seen as a case study for the germane tools and concepts, developed in analytic metaphysics, applied to modern physics. This illustrates not only their salient, and sometimes subtle, differences; it also shows the fertility of the cooperation between analytic metaphysics and philosophy of physics—and how thereby they can *mutually* constrain, and refine, each other’s insights.

We’ll proceed as follows. In Sect. 2, we review Lehmkuhl’s main, GR-based arguments, and explicate their key premises. In Sects. 3 and 4, we critically examine them from the perspective of philosophy of physics, and of metaphysics, respectively. These sections explore different ways of fleshing out and extend the core super-substantivalist commitment—different ways that we find problematic. This is important, we contend, because it paves the way for our positive proposal on how to reformulate and develop priority super-substantivalism in a way that steers clear of the aforementioned problems (Sect. 5).

## The super-substantivalist case from GR

In this section, we’ll first reconstruct Lehmkuhl’s characterisation of super-substantivalism (Sect. 2.1). We’ll then (Sect. 2.2) explicate his GR-based argument for super-substantivalism.

### Preliminary remark on terminology

According to Lehmkuhl (2018, p. 24; see also 2012), super-substantivalists subscribe to two tenets:

**(SUB-MON)** Substance Monism: There exists only one kind of substance.

**(ST-FUND)** Spacetime Fundamentalism: Spacetime is a fundamental substance.

Following the tradition of Aristotle, Descartes, Leibniz and Spinoza (cf. Robinson, nd), substances denote ultimate subjects of predication^4^: they are the ultimate bearers of properties; they are what, in the last instance, properties are, as it were, tacked onto.^5^ That is, every property can be *directly* attributed to spacetime, with no need for matter as an *extraneous* medium. Such substances needn’t be posited as independent entities in the manner of (bare) substrata. At this stage, we ought to construe the term maximally permissively: whatever entity (e.g. substrata, bundles of tropes or concrete things) plays the role of ultimate subject of predication, will do as a substance in the relevant sense.

Lehmkuhl further unpacks spacetime substantivalism, as just sketched, as a priority thesis: spacetime is supposed to be a *fundamental* physical substance in that it’s ontologically prior to matter. The “rough idea”, Lehmkuhl elucidates, is:

**(PRIORITY**_**0**_**)**: “[An entity] A is ontologically prior to [an entity] B *iff* the existence of A implies or contains the existence of B but not vice versa” (Lehmkuhl, 2018: 28)

With this taxonomy, Lehmkuhl succeeds in recovering how relationalism and substantivalism are commonly^6^ understood in the philosophy of physics literature. Significantly, it allows a demarcation of super-substantivalism from relationalism, as well as from substantivalism. Relationalists share with the super-substantivalist **(SUB-MON)**, but renounce her endorsement of **(ST-FUND)**. According to relationalism, all spatiotemporal talk in the last instance boils down to talk about spatiotemporal relations between material entities; material entities are the relata of spatiotemporal relations. Matter, on relationalism, is the only fundamental substance: spacetime structure is viewed as parasitic on it; it can’t exist without matter.

Substantivalists, on the other hand, share with the super-substantivalist **(ST-FUND)**; in contrast to her, however, they allow for a substance dualism by renouncing **(SUB-MON)**. *Both* matter and spacetime are assumed to be able to exist without the other.

We are now ready to address Lehmkuhl’s arguments for priority super-substantivalism.

### An argument for spacetime fundamentality from general relativity

For spacetime’s priority_0_ (in the sense of Sect. 2.1), Lehmkuhl propounds a two-pronged argument. It draws on contemporary gravitational theory—GR. We’ll dub the first part the “Energy–Stress Argument”. It emphasises that general-relativistic energy–stress presupposes the metric. Thereby, it seeks to show that the existence of matter implies the existence of spacetime. The second part we’ll dub the “Vacuum Argument”. It seeks to show that the converse doesn’t hold: spacetime can also exist in the absence of matter. If sound, both arguments establish the substantivalist’s core commitment to spacetime’s fundamentality (in the sense of **PRIORITY**_**0**_).

What makes Lehmkuhl’s argument enticing is that it rests on considerations of GR—a specific, extraordinarily successful physical theory. For the present purposes, basic facts about GR suffice. GR’s basic variable is the so-called metric $g_{ab} .$ For simplicity, we may think of it as a symmetric, $4 \times 4$-matrix-valued function (with $a,b = 0, \ldots ,3$) that generalises the Newtonian (scalar) potential $\varphi$. Its dynamical equations are the Einstein Equations—a set of 10 independent coupled differential equations:

$$ G_{ab} = \frac{8\pi G}{{c^{4} }}T_{ab} . $$

Here, $G$ and $c$ denote the gravitational constant and the speed of light, respectively. The (matrix-valued) object on the l.h.s., the so-called Einstein tensor$G_{ab}$, can be thought of as the expression of a non-linear differential operator (containing second derivatives), acting on the metric. It supplies the source-free dynamics for the metric. ($G_{ab}$ is thus the general-relativistic counterpart of the l.h.s. of Gauß’s Law of Newtonian Gravity, i.e. $\Delta \varphi : = div\left( {\vec{\nabla }\varphi } \right) = - 4\pi G\varrho$) The object on the r.h.s.—the so-called energy–stress tensor $T_{ab}$—acts as the source term for this dynamics. It’s constructed from the metric and the matter fields (e.g. electromagnetic fields). It earns its name from its direct connection with energy–stress, as familiar from pre-GR physics. Hence, it’s common to paraphrase the physical content of the r.h.s. of the Einstein Equations as the distribution of energy–stress of matter acting as the source for the dynamics for the metric (furnished by the l.h.s.).

Owing to the metric’s effects, GR is typically presented as a spacetime theory (see e.g. Friedman, 1983, Ch. 5; Maudlin, 2012, Ch. 6): the metric determines the time intervals surveyed by the ticking of clocks, as well as the lengths measured by rods. By the same token, it determines what counts as inertial/force-free motion in the resulting non-Euclidean geometry—the privileged path structure that is traced out by test-particles (via the general-relativistic counterpart of Newton’s First Law, see e.g. DiSalle, 2020). In this sense, in GR gravity manifests itself in effects of spatiotemporal geometry.

For spacetime to be prior_0_ to matter, whenever matter exists, spacetime must also exist. The Energy–Stress Argument attempts to demonstrate this. It turns on the energy–stress tensor’s dependence on the metric (cf. Lehmkuhl, 2011).

**(DEP)** The energy–stress tensor $T_{ab}^{{\Psi }}$ of a matter field ${\Psi }$ (as it features on the r.h.s. of the Einstein Equations) is defined (and receives its physical interpretation, ibid. Section 5) with respect to not only ${\Psi }$, but also with respect to the metric. In this sense, the energy–stress tensor presupposes the metric.^7^

The second premise declares the ascribability of an energy–stress tensor an *essential* criterion for materiality (see ibid.; cf. Martens & Lehmkuhl, 2020a, Sect. 3 for details):

**(MAT)** For a field to be a *matter* field ${\Psi }$ (rather than, say, a spacetime-geometric field), it’s essential that it possess an associated energy–stress tensor $T_{ab}^{{\Psi }}$.

**(DEP)** and **(MAT)** imply the (essential) dependence of matter on the metric: a matter field ${\Psi }$ eo ipso *presupposes* it.^8^ Without the metric-dependent energy–stress tensor, ${\Psi }$ would cease to represent a matter field; it could still exist simpliciter, though—but as a non-material field (e.g. a spacetime-geometric one).

Next, we identify the metric—or rather: the pair manifold-*cum*-metric ${\mathcal{M}},g$—with spacetime (cf. e.g. Hoefer, 1996; Maudlin, 1988, 1993):

**(INT-MET)** “the metric field $g$ is the referent of ‘spacetime (structure)” (Lehmkuhl, 2018, p. 33).

Let’s finally add the inference from essential dependence to *existential dependence* (more on this later on):

**(ES-EX)** Essential dependence entails existential dependence: if *x* is essential for *y*, then *y*’s existence depends on *x*’s.

With this, the preceding result entails that matter existentially depends on spacetime. This is necessary for the metric’s ontological **PRIORITY**_**0**_ over matter: insofar as the metric is *presupposed* by matter, the latter couldn’t exist without the former. This concludes the Energy–Stress Argument: together with **(DEP)**, **(MAT)**, and **(INT-MET)**, **(ES-EX)** implies that matter existentially depends on spacetime.

For **PRIORITY**_**0**_, it remains to be shown that the converse doesn’t hold. This the Vacuum Argument seeks to demonstrate. It comprises four premises. The first is a mathematical fact concerning GR’s solution space:

**(MATH-VAC)**: GR allows for vacuum solutions, i.e. solutions of the Einstein field equations without (non-gravitational) ordinary matter, such as fluids or electromagnetic radiation.

To distill from those vacuum solutions any metaphysical juice, we must ensure that at least some represent physically/nomologically possible worlds, i.e. genuine possible ways the world could be:

**(PHYS-VAC):** At least some matter-free vacuum solutions are physical: they denote real physical possibilities that should be taken seriously (rather than dismissed as merely formal, mathematical solutions, devoid of physical content).

Next, Lehmkuhl observes that the metric never takes on a zero value. In this sense, it vanishes nowhere; the structure it represents is always non-trivial:

**(NON-VAN)**: Even in vacuum solutions, the metric $g$ is present: nowhere does it vanish; it’s necessarily present (and non-trivial) in all models of GR (here equated with ‘possible worlds’, see, e.g., Pooley, 2013).

**(NON-VAN)** leaves unspecified *what* the metric represents. As does the Energy–Stress Argument, the Vacuum Argument assumes **(INT-MET)**: the metric represents *spatiotemporal* structure. After all, super-substantivalism is supposed to be a thesis about *spacetime*.

This concludes the Vacuum Argument: **(MATH-VAC)**, **(PHYS-VAC)**, **(NON-VAN)** and **(INT-MET)** imply that spacetime can exist even in the absence of matter, or independently of matter. Given **PRIORITY**_**0**_, the argument establishes priority super-substantivalism: spacetime is more fundamental than matter.

With this reconstruction in place, our next step will be to evaluate Lehmkuhl’s argument (and the prospects of using priority_0_). We’ll employ a division of labour: the next section (Sect. 3) will scrutinise Lehmkuhl’s arguments from the perspective of philosophy of physics, more narrowly construed; in Sect. 4, we’ll complement this by the perspective of analytic metaphysics.

## Critical assessment: philosophy of physics

This section will critically assess some of the premises of Lehmkuhl’s arguments from the philosophy of physics perspective. They turn out to depend on substantive interpretative assumptions; albeit not uncommon, these can, not implausibly, be questioned. Lehmkuhl’s argument is thus sensitively *conditional* on interpretative assumptions from the side of physics.^9^

First (Sect. 3.1), we’ll ponder whether Lehmkuhl’s arguments are confined to GR. We’ll then (Sect. 3.2) scrutinise **(MATH-VAC**), **(PHYS-VAC)** and **(INT-MET)** in the Vacuum Argument. Finally (Sect. 3.3), we turn to **(MAT)** in the Energy–Stress Argument.

### A comment on scope

Before taking a critical look at the Vacuum and the Energy–Stress Argument in greater detail, we’d like to *strengthen* them (recalling, and making explicit some remarks in Lehmkuhl’s (2011) earlier paper): neither depends on GR *specifically*; also, theories other than GR exemplify the features on which his arguments pivot.

The Vacuum Argument presupposes the existence of vacuum (matter-free) solutions. A relationalist will renounce **(PHYS-VAC)** in the case of absolute (non-dynamical) spacetime theories. In the case of *dynamical* ones, such as one encounters in GR, she might falter. Three reasons make it prima facie (more) plausible to countenance vacuum solutions as *physical*. The first is mutability—their generic dynamical nature: they evolve in time, independently of (i.e. not fully determined by) matter. Secondly, those degrees of freedom are contingent: they vary across the different models of GR. Thirdly, they are structurally rich, and give rise to complex novel phenomenology (e.g. gravitational waves or cosmic expansion). In analogy to other cases in physics (say the electromagnetic field or the quantum mechanical wavefunction), these features suggest that one take the metric’s degrees of freedom seriously on their own: they are plausibly to be viewed as “self-standing” entities that can be ascribed a robust physical status (cf. Brown & Wallace, 2005; Brown, 2005, Ch. 9).

If one accepts this reasoning, the Vacuum Argument applies to *any* dynamical spacetime theory admitting of vacuum solutions.^10^ (That is: supposing that the other premises remain intact.) Examples include Nordström’s theory of gravity in Einstein and Fokker’s spacetime-geometric formulation (see e.g. Norton, 1992; Dürr, 2019), or Cartan’s geometrised version of *Newtonian* Gravity^11^ (see e.g. Knox, 2014).

At the heart of the Energy–Stress Argument lies the recognition that the energy–stress tensor definitionally presupposes spacetime structure (represented by the metric). This feature, too, is shared by other theories (in agreement with Lehmkuhl, 2011): also in Newtonian Gravity, the energy–stress tensor presupposes the Newtonian spacetime structure (see e.g. Malament, 2012, Ch. 4). Likewise, the energy–stress tensor of a generic special-relativistic field depends on Minkowskian spacetime structure (cf. Lehmkuhl, 2011).

These observations are grist to the mills of Lehmkuhl’s argument: there are considerably more theories that support his arguments than just GR. By contrast, the remainder of this section and the next section will signal some problems with Lehmkuhl’s original argument. Yet, in Sect. 5, we’ll argue that the basic idea behind Lehmkuhl’s priority super-substantivalism can be developed in a propitious way. We offer this as a partial fulfilment of Lehmkuhl’s own understanding of priority super-substantivalism: we put some flesh on the bones of the basic idea he suggested. These details will avoid the problems we point out.

We’ll now evaluate the Energy–Stress and Vacuum Argument. Each rests on key assumptions that, upon closer inspection, turn out to be problematic.

### The vacuum argument revisited

Let’s scrutinise three premises of the Vacuum Argument—**(MATH-VAC)**, **(PHYS-VAC)** and **(INT-MET)**. All three turn out to have open flanks: they rely on non-trivial, interpretative *options* that turn out to be more less compelling than at first blush they might appear. As before, Lehmkuhl’s argument is a sensitively conditional one—as will please the naturalist.

According to **(MATH-VAC)**, GR (and in light of the above also other theories) admits of vacuum solutions (i.e. solutions in which matter is absent). Challenges to **(PHYS-VAC)** come from two directions: first by disputing a categorical matter/spacetime dichotomy, and secondly by pointing to the cosmological constant, interpreted as vacuum energy of an omnipresent field.

As explicated by **(NON-VAN)**, the metric $g$ vanishes nowhere. **(MATH-VAC), (PHYS-VAC)** and **(NON-VAN)** jointly imply that $g$ shouldn’t be classified as matter; else “vacuum” solutions wouldn’t be matter-free sensu stricto. The so-called “particle physics tradition” (Pitts, 2016) of GR opposes this consequence: Weinberg (1972, pp. 77, 147) or Rovelli (1997), for instance, interpret the metric simply as a matter field, responsible for gravitational effects. Such a matter interpretation of GR short-circuits the Vacuum Argument^12^: it renders “vacuum” solutions merely free of *non-gravitational* matter—but not of matter simpliciter. Construed literally, **(MATH-VAC) & (NON-VAN) & (PHYS-VAC) & (INT-MET)** would be false. (The same conclusion is reached, if one disputes the matter/spacetime distinction along more general lines—say, as merely conventional. We’ll revert to this thought in our discussion of **(INT-MET)** later on.)

**(PHYS-VAC)** can be attacked also from a more fundamental (quantum) perspective: due to quantum fluctuations, “vacuum” solutions cease to be matter-free in a strict sense; those fluctuations give rise to a positive cosmological constant, standardly interpreted as the “vacuum” energy of quantum fields permeating the entire cosmos. Cosmological evidence indeed strongly supports the existence of such a non-zero cosmological constant (see e.g. Carroll, 2001). As a result, the universe is filled with a plenum (the quantum fields): strictly speaking, again there are no vacuum solutions sensu stricto; **(PHYS-VAC)** becomes vacuous. Absent the modal qualifications in Lehmkuhl’s account (see Sect. 4), though, it’s difficult to gauge the force of this objection. And to be fair: Lehmkuhl only talks about classical/non-quantum physics. But this doesn’t quite alleviate the issue: the cosmological constant—introduced into the Einstein Equations on phenomenological grounds—can be interpreted as a “constant” energy contribution of a *classical*, omnipresent field that elicits a uniform, universal effect. Consequently, the possibility of a plenum also arises within classical relativistic field theory.^13^

Let’s move on to another premise of the Vacuum Argument—**(PHYS-VAC)**. Little seems problematic about it. A theory’s physically/nomologically possible worlds (see e.g. Fine, 2002) are given by its models (in the sense of the so-called semantic approach to theories, championed e.g. by Van Fraassen, 1980, 1989), i.e. suitable *n*-tuples of (geometric) objects that satisfy all of the theory’s axioms. As such, vacuum solutions of GR, evidently count as physically possible worlds.

Still, it’s fitting to broach a potential subtlety—Mach’s Principle. Roughly, it prescribes that inertia, and spacetime structure more generally, be explained dynamically via the interaction of physical/material bodies; spacetime and inertial structure are supposed to be “produced” by matter—rather than postulated as primitive givens. Conversely, absent matter, they should cease to be meaningfully defined. In the genesis of GR, Mach’s Principle played a crucial, historical role (see e.g. Hoefer, 1994, 1995). In some formulation or other, it has persistently arrested physicists’ fascination (see Torretti, 1984, p. 202 for references; Bondi & Samuel, 1996; King & Pfister, 2014). Ultimately, it had to be abandoned (see e.g. Torretti, 1984, p. 199; Brown & Lehmkuhl, 2013).

An attenuated reading of Mach’s Principle, however, prima facie fares better: indeed, it seems compatible with GR in *all relevant* applications—but bans global vacuum solutions as unphysical. Mimicking Einstein’s initial reaction to the pressure under which his original vision of Mach’s Principle had come in the debate with de Sitter (Janssen, 2013; Sect. 5; Lehmkuhl, 2014; Sect. 2.1), one may be tempted to append it to GR’s axioms as a selection rule for *physical* spacetimes: compliance with it then demarcates physical from unphysical, merely formal solutions.^14^ The selection rule in question would be a causality constraint, broadly construed:

**(MP-WEAK)**: Spacetimes must contain matter-filled regions.

In contrast to the standard historical form of Mach's Principle (according to which, roughly, the matter distribution is supposed to fully determine—be a sufficient reason for—spacetime), (MP-WEAK) *doesn't* require that matter fully determine spacetime structure. Rather, (MP-WEAK) is a metaphysical selection principle—a necessary condition—for physical spacetimes. One may view (MP-WEAK) as reflecting a tight, law-like relation between matter and spacetime—a relation, however, weaker than determination. Note that the principle still allows for gravitational wave solutions. This includes *all* the standard ones, discussed in astrophysical practice—barely a surprise: astrophysics is concerned with gravitational radiation emitted by *material sources*, e.g. neutron stars, or black holes. Also, the Schwarzschild and the Kerr solution, decent models of isolated, (non-) rotating stellar objects abide by (MP-WEAK)—provided one attaches to them an interior, matter-filled solution. (MP-WEAK) only weeds out *entirely* matter-free solutions (such as Weyl’s static, axisymmetric vacuum solutions, or the Gowdy universe, filled only with gravitational radiation).^15^

Our point isn’t necessarily to endorse (MP-WEAK). Interestingly, however, Norton’s (ms) recently proposed empiricist conception of modality, underwrites (MP-WEAK): globally vacuum spacetimes *are* ruled out as (physically) impossible. We merely point out that at first blush, such a principle—a square challenge to (PHYS-VAC)—isn’t altogether devoid of plausibility. To the extent that the Vacuum Argument requires (PHYS-VAC), it’s therefore desirable that the loophole, opened by (MP-WEAK) (or a similar principle), be foreclosed.

Finally, **(INT-MET)** deserves a comment. The interpretation of the metric as spatiotemporal is a crucial premise: without it, the Vacuum Argument (as well as the Energy–Stress Argument) remains silent about spacetime; even if otherwise sound, it would only entail that the metric can exist without ordinary matter. But super-substantivalism is supposed to be thesis about the priority of *spacetime*. Whether we should interpret the metric as spatiotemporal is indeed controversial, as Lehmkuhl himself notices (see e.g. Lehmkuhl, 2008, 2014; Rey, 2013).

At least two alternatives must be reckoned with. The first construes the metric as a universal force-field (as defined by Reichenbach, see e.g. Carnap, 1957, Sect. 6): it’s a gravitational field that *also* affects rods and clocks. On this interpretation, the metric represents gravitational matter; it’s not *inherently* tied to spacetime. If otherwise sound, the Vacuum and Energy–Stress Argument would, were **(INT-MET)** replaced with this universal force-field interpretation, entail the priority_0_ of gravity (gravitational matter) over non-gravitational matter—a position evidently distinct from super-substantivalism, as introduced in Sect. 2.1.

Another challenging alternative to **(INT-MET)** stems from conventionalism about geometry, as historically originating with Poincaré (see e.g. Ben-Menahem, 2001; Ivanova, 2015): according to conventionalism, talk about spacetime geometry doesn’t correspond to any facts; geometric facts are merely conventional stipulations—neither empirical nor analytic truths (for details, see e.g. Ben-Menahem, 2006; Pitts, 2016; Dürr, 2021). Such conventionalism undercuts **(INT-MET)**: conventionalism divests it of physical content. By the same token, super-substantivalism’s core commitment to *spacetime’s* fundamentality would fade away into conventionality.

Here, we are non-partisan vis-à-vis these interpretative stances towards the metric. The lesson, however, for the status of **(INT-MET)** is clear: it’s a substantive assumption that requires independent arguments—or at any rate is a substantive *input from* (philosophy of) *physics.*

In short: As it stands, the Vacuum Argument is best construed as a conditional conclusion: it’s predicated on non-trivial premises, in particular, concerning the interpretation of the metric as spacetime, i.e. **(INT-MET)**, and the physicality of global vacuum solutions, i.e. **(PHYS-VAC)**.

### The energy–stress argument revisited

The Energy–Stress and Vacuum Argument share **(INT-MET)** as a premise. Objections to **(INT-MET)** hence impinge upon both. Here, we turn to the other crucial premise in the Energy–Stress Argument, **(MAT)**.

Should we believe that it’s essential for a matter field to assign it an energy–stress tensor, as **(MAT)** asserts? The following thoughts cast doubt on this.

It will be convenient to decompose **(MAT)** into two components:

**(MAT**_**1**_**)** Possessing energy-stress constitutes (part of) the essence of matter. Conversely, an entity lacking energy-stress is necessarily immaterial (e.g. a number, a law, a universal, etc.).

**(MAT**_**2**_**)** In GR, the energy–stress tensor $T_{ab}$ rightly bears its name: first and foremost – *essentially* – it represents the associated field’s energy–stress.

Both sub-tenets of **(MAT)** are problematic. Let’s commence with **(MAT**_**2**_**)**. One may forthrightly gainsay: despite its label, it’s plausible to claim that $T_{ab}$ *doesn’t* primarily represent energy–stress. Following e.g. Schrödinger (1950, p. 99), it has indeed been argued (e.g. Pitts, 2016, Sect. 2; Dürr, 2018, Sect. 3.4) that first and foremost $T_{ab}$ denotes the source term for the Einstein Equations. (Such an interpretation accrues further support from the analogy with Yang-Mills theories, i.e. theories which successfully describe the other fundamental forces in elementary particle physics.) Energy–stress proper is defined via Noether currents, associated with rigid spacetime translations—in line with the standard view in field theory (see e.g. Schmutzer, 1972). One therefore shouldn’t conceptually *identify* $T_{ab}$ and energy–stress proper. In fact, they can come apart (see e.g. Leclerc, 2006, Sect. 2 for an explicit example, cf. Szabados, 2012, Ch.2.1). Notwithstanding their conceptual distinctness, they coincide in certain (sufficiently symmetric) spacetimes (perhaps even in generic ones, up to some degree of approximation, cf. Fletcher, 2019).

Due to its overtly metaphysical nature, we’ll delegate the bulk of our discussion of **(MAT**_**1**_**)** to Sect. 4. Also here questions arise, however, from the perspective of philosophy of physics. GR isn’t the last world on spacetime or gravity: almost certainly, it will be superseded by a future theory that incorporates quantum effects. Hence, it strikes us as bold to tie a claim about the essence of matter to *classical* GR’s energy–stress tensor. Interestingly, Einstein himself harboured scepticism about the energy–stress tensor: he regarded it merely as a *provisional* placeholder—a phenomenological element to be replaced by a more fundamental, future quantum theory of matter (Lehmkuhl, 2019). Shouldn’t this curb our confidence in **(MAT**_**1**_**)**?^16^

Indulgence in a little speculation fuels further suspicion—even at the *classical* level. Consider theories *wildly* different from GR: theories may well exist (even if empirically inadequate) whose treatment of the (classical) matter sector doesn’t involve a metric, or in which multiple metrics exist.^17^ In such theories, the standard definition of the energy–stress tensor, familiar from GR (viz. as a variational derivative with respect to the metric), doesn’t straightforwardly carry over. The same conclusion holds for theories that don’t possess a Lagrangian formulation (with the energy–stress tensor being defined as the variational derivative of the Lagrangian).^18^ Should we conclude that such theories don’t contain matter? That would seem a bit rash. A more plausible conclusion, we think, would be to demand that claims about the essence of matter should be modally robust enough (and not merely confined to worlds with the same laws as ours, for otherwise the insistence on the *essence* of matter would hardly be justified) so as to yield illuminating answers also beyond the realm of GR. That **(MAT**_**1**_**)** flouts this desideratum may, conversely, be deemed a reason to question its adequacy.

In conclusion: the Energy–Stress Argument stands on controversial premises; in addition to open questions regarding the metric’s spacetime interpretation, one may query both the identification $T_{ab}$ as energy–stress proper, and the existence of an energy–stress tensor as an essential criterion for materiality. An explicit recognition of the controversial nature of its premises seems to us necessary for a proper evaluation of the arguments in favour of priority super-substantivalism.

In this section, we analysed Lehmkuhl’s reasoning from the vantage point of philosophy of physics. Its premises were shown to be less innocent than their common endorsement might suggest. We argued, (at least) some of them stand in need of independent justification; not implausibly, one may in fact squarely challenge them. But even if one ultimately accepts those premises, our results yield something of interest: for Lehmkuhl’s argument to go through, substantive interpretative assumptions on the side of physics are indispensable; that is, his metaphysical argument for super-substantivalism relies on physics even more sensitively than initially thought. This will please friends of “naturalised metaphysics” (such as Ladyman & Ross, 2007). In the same vein, it will also be grist to the mills of Lehmkuhl’s professed preference for metaphysical positions that physical considerations can impel us to forsake. (More on this in Sect. 4.3.)

With super-substantivalism being a metaphysical thesis about fundamentality, the next task is to supplement the foregoing analysis by one from the perspective of analytic metaphysics.

## A critical assessment: metaphysics

This section will direct attention to the key metaphysical notions in super-substantivalism’s profile as a fundamentality/priority thesis. A critical assessment of Lehmkuhl’s original proposal from the metaphysical perspective will serve as a foil. Our principal point of criticism is that asymmetric existential dependence, as it features in Lehmkuhl’s reasoning, *doesn’t* track fundamentality. We discuss whether other—kindred, yet distinct—forms of ontological dependence are better suited. Section 4.1 will hone in on ontological dependence and its connection with fundamentality (Sect. 4.1). In Sect. 4.2, we’ll turn to recent proposals for relative fundamentality (Sect. 4.2). Section 4.3 will remark upon the link between relative and absolute fundamentality.

### Ontological dependence

Here, we’ll elaborate different worries about **PRIORITY**_**0**_ *even if viewed as a “rough idea”*: the first concerns relevant modal aspects; the second targets that rather than capturing fundamentality/priority, **PRIORITY**_**0**_ amounts to *ontological dependence*—a *distinct* notion.

Two observations are apposite. First, the link between priority and ontological dependence (which Lehmkuhl himself, quoting seminal works on ontological dependence, e.g. Fine, 1995 and Correia, 2008, directly relates), turns out to be less innocent than one might think, as we’ll see presently below.

Second, modal considerations are crucial for the Vacuum Argument: it considers solutions that are countenanced as *possible but not actual worlds*. Our world isn’t a vacuum world. Hence, in order to gain metaphysical mileage from the existence of vacuum worlds as formal solutions of GR, any characterisation of ontological priority undergirding **(PRIOR**_**0**_**)** must incorporate modal aspects. Thus, a more perspicuous formulation of **PRIORITY**_**0**_ would be:

**(Ont-Prior)**: *x* is ontologically prior to *y* =_def_ Necessarily [if *y* exists then *x* exists], but not vice-versa.

The very formulation of **(Ont-Prior**) is important because it renders transparent that the target notion *isn’t* ontological priority/fundamentality per se; rather, it’s asymmetric existential dependence—*definitionally* equated with fundamentality. To see this, note that, apart from the anti-symmetry conjunct (i.e. “not vice versa”), **(Ont-Prior)** tallies verbatim with the definition of dependence—not priority—in the so-called *Simple Modal Account* of dependence (Fine, 1995 and Correia, 2008).^19^

Recall that (following Lehmkuhl) we introduced super-substantivalism as a priority (fundamentality) thesis. But fundamentality and ontological dependence are distinct notions (more on this in a moment). Any *definitional* identification, such as the one in **(Ont-Prior)**, is hence problematic. Once one recognises this, it’s immediate to see that, in their original form, Lehmkuhl’s arguments can’t buttress super-substantivalism: at best, they establish that matter asymmetrically depends on spacetime. For this to support super-substantivalism, we need a connection between ontological dependence and priority. This is ensured by the following conditional:

**(Dep → Ont-Prior)**: If *x* ontologically depends on *y*, then *y* is ontologically prior to (more fundamental than) *x*.

This we suggest as a first friendly amendment to Lehmkuhl’s discussion: distinguish dependence and priority, and then use something like the conditional above. In effect, with this in place, the Vacuum and Energy–Stress Arguments establish what they are supposed to: if otherwise sound, given **(Dep → Ont-Prior)**, they imply the priority of spacetime—as the super-substantivalist asserts.

But now we may wonder: how convincing is **(Dep → Ont-Prior)**? This we’ll now assess. The remainder of this section will zoom in on the relevant notion of dependence in the antecedent of **(Dep → Ont-Prior)**; in Sect. 4.2, we’ll take a closer look at the notion of relative ontological priority in the consequent. To foreshadow our conclusion: the viability of **(Dep → Ont-Prior)** depends on *crucial details* of both the relation of dependence, and that of ontological priority; a “rough idea” simply isn’t enough.

A natural place to start is the Simple Modal Account of dependence. As we saw, this is operative in **PRIORITY**_**0**_ and **Ont-Prior**. Insofar as one relies on it, the case for super-substantivalism is compromised: a number of well-known drawbacks beset the Simple Modal Account. In the case at hand, they are especially problematic.

One involves necessary objects—i.e. objects whose non-existence is impossible (to the effect that conversely, they must necessarily exist, i.e. exist in all possible worlds). Suppose that they exist—say, mathematical objects, e.g. sets or functions. The Simple Modal Account then implies that every contingent (i.e. non-necessary) entity asymmetrically depends on any such necessary object: the existence of every contingent object necessitates the existence of every necessary one; but the converse doesn’t hold. That is: in every possible world in which *some* contingent entity exists, also *any* necessary object exists; but not vice versa.

One may deem this already rebarbative in and of itself. In the present context, the issue furthermore threatens one of the core commitments of super-substantivalism—**(ST-FUND)**, i.e. the thesis that spacetime is a fundamental substance. Here, a fundamental substance presumably isn’t supposed to depend on anything else (see e.g. Tahko & Lowe, 2015, Sect. 6.3).^20^ Such an understanding of fundamental substances indeed follows from **(Dep → Ont-Prior)**, when conjoined with the (plausible) assumption that something is fundamental iff nothing is more fundamental than it (see Sect. 4.3). Then, if necessary objects exist, spacetime doesn’t qualify as an independent, fundamental substance—in conflict with the super-substantivalist’s commitment to **(ST-FUND)**.

An immediate counter to defuse this objection is to deny the existence of necessary objects. Clearly such a denial requires independent arguments, but there are some compelling ones. This however won’t be enough to rescue the account at hand. This is mostly because, even when suitably hedged via the asymmetry clause, the Simple Modal Account fails to track relative fundamentality.^21^ In other words, if dependence is constructed along purely modal lines, it fails to support the principle **(Dep → Ont-Prior)**.^22^ Let’s quote Correia (2008, p. 11) at length:

Can we say that a claim of type ‘*a* rigidly necessitates *b*’ [i.e. whenever *a* exist, necessarily so does *b*; our addition] conveys the idea that the existence of a is derivative upon, or less fundamental than, the existence of b, in some sense of ‘derivative’ or ‘fundamental’? Hardly so. For the intended relation of existential derivativeness must arguably be irreflexive, and even asymmetric, while rigid necessitation is reflexive and (therefore) not asymmetric.

In other words, Correia’s critique animadverts upon the entailment between (relative) fundamentality and modal existential dependence simpliciter. Does the requirement of *asymmetric* existential dependence help? Unfortunately, it doesn’t. Correia continues:

What about one-way rigid necessitation, i.e. the asymmetric relation an object x bears to an object y when x rigidly necessitates y but not vice versa? It does not capture the idea of existential derivativeness either. For instance, it is plausible to hold that Socrates is a contingent existent and that the empty set is a necessary existent. But that view implies that Socrates one-way rigidly necessitates the empty set, and we do not want to say that the existence of the former is derivative upon that of the latter. Further arguments for the same conclusion can be formulated by invoking types and lives instead of necessary existents (Correia, 2008, p. 11).

Correia’s last passage illustrates that the failure of existential dependence—at least when construed along purely modal lines—to track relative fundamentality can't be remedied simply by rejecting necessary existents. As Correia intimates, it’s plausible to assume that, for instance, Socrates rigidly necessitates the existence of the type ‘human being’. That is: in every world in which some particular entity exists, the type of which it’s a token exists, as well; the converse, doesn’t hold, though. Yet, this asymmetric dependence arguably doesn’t track fundamentality: tokens—despite asymmetrically depending on their types—are usually *not* viewed as less fundamental than their types.

Unless principled arguments are forthcoming to block such cases, an inference from (asymmetric) existential dependence to fundamentality, seems unwarranted. It calls for a substantive, independent justification.

In response to the problems of the Simple Modal Account, alternative accounts of ontological dependence have been put forward that might be harnessed more successfully in the context of fundamentality: are these alternative accounts more apt to underpin something like **(Dep → Ont-Prior)**? A widespread proposal rests on *essential dependence* (Fine, 1995). Fine argues that purely modal connections between the existence of two entities are too weak to do justice to claims of dependence. Instead, one should tie the necessity of the conditional expressing the dependence of *x* on *y* to the essence/nature of the dependent entity *x*: for *x* to depend on *y*, it must lie in *x*’s essence that it exists only if *y* exists.

Appeal to essential dependence in this sense eschews both problems of the Simple Modal Account. It doesn’t deliver the problematic result that any contingent entity depends on any necessary one: it insists on a more intricate, *essential* connection between the dependent and the dependee. Prima facie, essential dependence intuitively also seems to track relative fundamentality: if *x*’s existence ‘flows’ from *y*’s nature, to use Fine’s (1994, p. 9) suggestive phrase, (rather than merely accompanies’s existence, albeit in a modally robust manner, as in the above case of modal dependence), *y* may indeed be said to be prior to *x*.^23^

Yet, this solution comes at a price. In particular, some friends of naturalised metaphysics are likely to baulk at essentialism; in particular, those contesting the existence of essences will dismiss the account tout court.^24^

One may therefore wish to consider yet another account of ontological dependence, *explanatory* dependence (see e.g. Correia, 2005 and Schnieder 2006): *x* depends on *y* iff necessarily some feature *F* of *y* explains the existence of *x*. Typically, such an explanation is predicated on a particular relation—*grounding*.^25^

Suppose that one finds explanatory dependence an auspicious candidate relation of ontological dependence that tracks relative fundamentality. (That is, suppose that that **(Dep → Ont-Prior)** is licensed, with the relevant form of ontological dependence being explanatory dependence.) Accordingly, as in the previous case of essential dependence, let’s substitute “ontological dependence” in the Vacuum and Stress Energy arguments by “explanatory dependence”. Next, we must ponder whether the arguments go through: does GR support the claim that existence of matter is grounded in some feature *F* of spacetime? If so, what is that feature *F*?

Lehmkuhl’s original arguments remain silent on these questions. We’ll return to this in Sect. 5. In fact, we’ll maintain that explanatory dependence (upon filling in some prerequisite details) is the best candidate to underwrite the super-substantivalist argument. But before getting to that point, we must first gain greater clarity regarding the notion of fundamentality.

### Ontological priority

The previous subsection focused on the notion of dependence figuring in the antecedent of **(Dep → Ont-Prior)**. We argued that **(Dep → Ont-Prior)** depends on significant details about the notion of dependence; whether such details support the original arguments is (at best) unclear.

Here, we’ll look at the crucial notion in the consequent of **(Dep → Ont-Prior)**—namely, relative priority/fundamentality. Our conclusion is similar to that of Sect. 4.1: whether **(Dep → Ont-Prior)** holds hinges on the account of fundamentality/priority one adopts. Without a more detailed account of (relative) fundamentality, one can’t instructively evaluate the principle, nor arguments that depend on it.

To substantiate this claim, we’ll inspect two accounts of relative priority. On the first, it’s *unclear* whether **(Dep → Ont-Prior)** holds. On the second, it *fails*, at least in its generality.

Let’s start with Bennett’s (2017) account of priority. She maintains that relative priority is tracked by what she calls *building relations*. That is, for any building relation *R*:

**(Build R → Ont-Prior)**: If *x* is *R*-related to *y*, then *y* is ontologically prior_R_ to *x*.

Equivalently, if *x* is *partly built* by *y*, *y* is prior_R_ to *x*. (Notice the indexing, i.e. the *R*-relativity of Bennett’s notion of priority.) This seems close enough to **(Dep → Ont-Prior)**. One may therefore be hopeful that Bennett’s account vindicates the principle on which Lehmkuhl’s super-substantivalist arguments pivot. To see to what extent this hope is borne out, two issues must be addressed.

First, for **(Dep → Ont-Prior)** to count as an instance of (**Build R → Ont-Prior)**, we must argue that ontological dependence is a building relation. Bennett lists several examples for candidate building relations: e.g. composition, constitution, set-formation, realisation, micro-based determination, and grounding. Ontological dependence isn’t mentioned explicitly. Should we include it?

According to Bennett, a

(r)elation R is a building relation if and only if:

(1) For all *x*, ~R*xx*, and

(2) For all *x* and y such that *x* ≠ *y*, if *x* R *y*, then ~(*y*R*x*), and

(3) Let C be some to-be-specified set of background circumstances that

includes neither *y* nor anything that fully builds *y*. For all *x* and *y*, if *x*

fully R’s *y*, □[(*x* + C) → *y*]. [That is: necessarily, given the circumstances *C*, *x* entails *y*, our addition.]

(4) For all *x* and *y*, *x*’s R-ing *y* licenses explanatory and generative claims to

the effect that *y* exists or obtains in virtue of *x* (Bennett, 2017, p. 60).

Does ontological dependence—in *any* of the characterisations discussed in the previous section (i.e. existential, essential or explanatory ontological dependence)—qualify as a building relation? The question is non-trivial; its answer doesn’t seem straightforward. (Note, however, that the explanatory claim in condition (4) is most easily met by explanatory dependence. This will partly justify our argument in Sect. 5.)

A second challenge to applying Bennett’s account to Lehmkuhl’s case for super-substantivalism lies in the *R*-relativity of her notion of priority: the relation of ontological priority in the consequent of **(Build R → Ont-Prior)** is indexed to a particular relation *R*. Accordingly, different building relations *R* can induce different notions of ontological priority (i.e. priority_*R*_).

At this juncture, an advocate of Lehmkuhl’s arguments for super-substantivalism can pursue two different strategies. Either she has to show that the notion of fundamentality (priority) *relevant* for super-substantivalism is the one indexed to the form of ontological dependence one chooses (i.e. existential, explanatory, or essential). Alternatively, she may accept that one should distinguish between various forms of super-substantivalism, corresponding to the various notion of (dependence-relative) priority. (Apart from the usual objections to such constructions along the lines of gerrymandering, in principle, nothing forbids a disjunctive characterisation of super-substantivalism’s fundamentality thesis: spacetime must be fundamental (prior) with respect to *at least one* notion of dependence-relative priority.) Perhaps this second option is more in line with the original proposal by Lehmkuhl himself: he indeed points out that different specifications of the notion of ontological priority could give rise to different versions of priority super-substantivalism.

Once again, our point *isn’t* that **(Dep → Ont-Prior)** is false. We merely stress that its evaluation requires substantive details about the account of ontological priority one adopts.

Another account of fundamentality underscores this lesson even more: on that account, the principle **(Dep → Ont-Prior)** comes out as false. The account we have in mind is a particular example of a family of accounts which Correia (ms.) calls *Categorical Accounts*. The principal idea builds on the pyramid view of reality, intuitive and familiar to many physicists: reality has a stratified, hierarchical structure, composed of *levels* of different relative fundamentality. *Entities* belonging to a more fundamental level are more fundamental than entities belonging to a less fundamental one. As an illustration consider the following passage from a standard physics introduction to the standard model of particle physics^26^:

As was already briefly mentioned, hadrons (protons, neutrons, pions…) are built up from quarks bound by gluons. The QCD force between particles with color charge binds them into hadrons. The residual color forces outside color neutral hadrons is the nuclear force, that binds stable hadrons into nuclei. The electrically charged nuclei and stable electrically charged leptons (only the electron) are bound into atoms by the electromagnetic force, mediated by photons. The residual electromagnetic force outside electrically neutral atoms binds them into molecules. Thus the hierarchy of structures in nature is built. (Kane, 1993, p. 10)

For concreteness, consider a slightly simplified explication.^27^ Consider any set of entities *S*. Now impose a set-theoretical partition on *S* that divides *S* into subsets, called *Levels*, such that the levels are pairwise disjoint, and their union is *S*. A naïve example exclusively for the sake of illustration might help. Say that *L*_*1*_ is the level of physical entities, *L*_*2*_ of chemical entities, *L*_*3*_ of biological entities, *L*_*4*_ the level of social entities and so on.

One now defines a notion of ontological priority *between levels* as follows:

**(Level-Prior)** Level *L*_*i*_ is ontologically prior to level *L*_*j*_ iff for any entity *x* in level *L*_*j*_ there is an entity *y* in level *L*_*i*_ such that *x* depends on *y*.

Next, one defines a notion of ontological priority *between entities* as follows:

**(Entity-Prior)** *x* is ontologically prior to *y* iff *x* belongs to *L*_*x*_ and *y* belongs to *L*_*y*_ and *L*_*x*_ is ontologically prior to *L*_*y*_, where priority of levels is construed in terms of **(Level-Prior)**.

Nothing in this level-based approach to fundamentality rules out possible dependence relations between entities at the same level. Our toy example demonstrates this: dependence relations between purely, say, biological entities aren’t implausible. For example, mammals depend on various organs, such as the heart.^28^ In the present context, this would invalidate **(Dep → Ont-Prior)**: if an account of fundamentality allows for intra-level ontological dependence relations, **(Dep → Ont-Prior)** is false on this account.

To reiterate: we *don’t* claim that this shows that the original super-substantivalist arguments are fatally flawed. First, the categorical account of fundamentality isn’t uncontroversial. Secondly, it’s conceivable that one could re-work some of details of the account to make it fit with the said arguments. (For instance, one could start by envisaging a partition such that spatiotemporal entities and material entities belong to different levels. If spacetime and matter belong to different levels ab initio, the account guarantees that one is prior to the other; the super-substantivalist arguments might then be taken to show that the direction of relative priority is indeed from spacetime to matter.) Rather, our point is that the super-substantivalist arguments’ fate crucially depends on substantive details about how ontological priority is construed. In this regard, a super-substantivalist must put her cards on the table.

### Relative and absolute ontological priority

The super-substantivalist arguments seek to establish that spacetime is ontologically prior to matter. Yet, even if convincing, this wouldn’t *quite* suffice to vindicate super-substantivalism, construed narrowly as asserting spacetime’s absolute fundamentality. For that, we’d still need an inference from relative (“more fundamental than”) to *absolute* fundamentality (fundamental simpliciter); super-substantivalism is a claim about the latter.

It’s not difficult to see how to pass from relative to absolute claims of fundamentality. For instance, it’s plausible to define Absolute Priority from Relative Priority as follows:

**(Abs-Prior)**: *x* is absolutely prior (or absolutely fundamental), iff there is no *y* such that *y* is ontologically prior to *x*.

Suppose that Lehmkuhl’s *classical GR*-based arguments for spacetime’s absolute fundamentality succeeded. While this would establish super-substantivalism in the classical domain, it wouldn’t necessarily establish super-substantivalism, narrowly construed—as asserting spacetime’s unqualified absolute fundamentality; spacetime’s absolute fundamentality in the classical realm is compatible with its non-fundamentality at a deeper, quantum level. In fact, an entire research programme—what Martens (2020) calls the ‘Emergent Spacetime’ research programme—seems (or at least, seeks) to show that spacetime is derivative and non-fundamental, once one moves *beyond classical GR* (see e.g. Wüthrich, 2017, 2018; Wüthrich & Huggett, 2018). A successful Emergent Spacetime research programme would further curtail the force of the arguments for super-substantivalism discussed here: they would only establish the ontological priority of spacetime over matter (i.e. classical spacetime’s *relative* fundamentality); they wouldn’t establish the truth of super-substantivalism at a deeper, post-classical level.^29^

Dialectically, this might be important. In his paper, Lehmkuhl deplores that what he calls modest super-substantivalism—roughly coinciding with what we labelled “identity super-substantivalism”’ (Sect. 1)—is a

(p)urely metaphysical standpoint that can be taken quite independently from the physical theory we find to be true (…). Not much can happen to the modest super substantivalist, neither good nor bad things: however physics develops, there is a way for him to uphold his position (Lehmkuhl, 2018, pp. 38–40).

The aforesaid, we believe, ought to assuage Lehmkuhl’s disappointment with modest super-substantivalism: if physical developments were to point towards the ontological derivativeness of spacetime, this will affect all varieties of super-substantivalism that endorse **(ST-FUND)** in Sect. 2. Insofar as identity super-substantivalism and priority super-substantivalism both endorse **(ST-FUND),** they can both scupper on physics.^30^ We welcome such sensitivity to physical developments as good news—in full agreement with Lehmkuhl.

To take stock: both from the perspective of philosophy of physics, as well as from the perspective of analytic metaphysics, one may harbour doubts about the original super-substantivalist proposal. Lehmkuhl himself seems to think of it as providing only the bones, the skeleton of priority super-substantivalism. In the next section, we’ll first mend some broken bones and then put some flesh on them. In doing so we’ll provide what we think is the strongest case for priority super-substantivalism, a case that turns out to be (to our minds) convincing—and naturally in line with a common understanding of the role and status of spacetime structure in physics.

## Putting flesh on the bones: a better super-substantivalist argument?

Hitherto, our discussion has been somewhat negative: primarily, we alerted the reader to various drawbacks in Lehmkuhl’s original formulation of the main argument for super-substantivalism. We read the arguments in the previous sections as paving the way to a *positive* way of cashing out priority super-substantivalism. This will be provided here.

In Sect. 4, we saw that the inference from ontological dependence to priority/fundamentality—the principle **(Dep **$\to$ **Ont-Prior)**—puts constraints on both the notion of dependence, and that of ontological priority. Below, we’ll discuss three candidate relations of dependence between spacetime and matter: explanatory dependence and full and partial^31^ grounding. We’ll argue in favour of explanatory dependence. This salvages **(Dep **$\to$ **Ont-Prior)**—to the effect that the super-substantivalist’s argument for spacetime’s fundamentality goes through.

Considering explanatory dependence and grounding is driven by two kinds of motivations. First, explanatory dependence in principle looks apt to track relative fundamentality—at least, in some of the accounts of relative fundamentality we reviewed, viz. Bennett’s. As for grounding, Bennett herself includes it in her list of building relations. So, at least within Bennett’s account of priority, the counterpart of **(Dep → Ont-Prior)**, **(Ground → Ont-Prior)**, seems warranted:

**(Ground → Ont-Prior)** If *x* is *grounded* in *y*, then *y* is prior than *x*.

Secondly, both ontological dependence and grounding underwrite particular *explanations:* viz. non-causal, metaphysical explanations. At least on a common view on the role of spacetime geometry, spacetime structure metaphysically explains certain aspects of matter. More on this shortly.

Let’s therefore moot the following three claims about spacetime’s dependence on matter:

**(EXP-DEP-ST)** Matter explanatorily depends on spacetime. That is (cf. Correia, 2005): necessarily, there is something about spacetime, its being F, that partially grounds the existence of matter.

**(GROUND-PART)** The existence of spacetime *partially* grounds the existence of matter.^32^

**(GROUND-FULL)** The existence of spacetime *fully* grounds the existence of matter.

Let’s start with **(GROUND-FULL)**. If one endorsed somewhat orthodox principles about grounding, namely necessitarianism (i.e. the view that if *x* grounds *y*, necessarily *x* entails *y*),-one would undermine the Vacuum Argument. This is because the existence of spacetime would necessitate the existence of matter. That is, in any world in which there is spacetime, there is matter—contra the Vacuum Argument. This leaves us with **(EXP-DEP-ST)** and **(GROUND-PART)**. Partial grounding figures in both. Yet, their distinctness must be stressed. **(EXP-DEP-ST)** purports strong modal connections between matter and spacetime: in every world in which there is matter, there is something about spacetime—some *F*—that metaphysically explains the existence of matter. Given that *F* is usually taken to be existence-entailing (more on this shortly), it follows that, according to **(EXP-DEP-ST)**, in every world in which matter exists, spacetime exists. By contrast, **(GROUND-PART)** lacks such modal ramifications: **(GROUND-PART)** per se *doesn’t* enforce any modal connection between spacetime and matter.

This distinction becomes important for some super-substantivalist arguments. We already saw that modal considerations are integral to Lehmkuhl’s main argument. A key one is the deliberation whether spacetime exists in every world in which matter exists. This suggests that explanatory dependence—rather than partial grounding—is the best relation to underwrite such arguments.

Explanatory dependence has the right modal force; by contradistinction, partial grounding has *none*. Likewise, explanatory dependence has the right modal direction; full grounding has the *wrong* one. Fully grounding entails that every world in which there is matter there is spacetime, whereas the converse does not hold.

This also addresses a possible objection: one might worry that explanatory dependence is *too weak* to secure super-substantivalism, since partial grounding requires something more than spacetime to get matter, so to speak. For a reply one needs only assume that *F* is existence-entailing—an assumption that is taken to be inherently plausible in the literature. For instance, Correia (2005, p. 70) writes:

[A] constraint is imposed on the feature of the base for it to be a base [i.e., the set of properties *F* that partially ground the existence of the dependent entity]. The constraint is that the feature be ‘‘existence-entailing’’, in the sense that having that feature requires existing.

If *F* is existence-entailing, it turns out that our proposal is *strictly stronger* than Lehmkuhl’s: our proposal entails his, but the converse doesn’t hold. (In effect, many cases of modal existential dependence don’t qualify as cases of explanatory dependence.) Hence, *if* Lehmkul’s proposal counts as Priority Supersubstantivalism, so does ours.^33^

But *does* matter explanatorily depend on spacetime—in the sense that necessarily, its existence is partially grounded in some spatiotemporal features? The received, so-called geometric approach to spacetime (canonised as orthodoxy for GR by Misner et al., 1973) can be construed as affirming this.

In what follows, we’ll unravel this thought in three steps: first, by substantiating the claim that spacetime structure can (on GR’s geometric interpretation) explain some material phenomena; secondly, by making it plausible that such geometric explanations are non-causal, and reasonably similar to paradigmatic examples of metaphysical explanations; and thirdly, by showing that Lehmkuhl’s argument actually goes through for explanatory dependence.

Let’s begin with the status of spacetime structure as an explanans (at least in some contexts). Why is Mercury’s orbit rosetta-shaped rather than a Keplerian ellipse? Why does a radar signal passing by a planet take longer for its journey than through the void? Why do gyroscopes precess in the presence of a massive, rotating body? Why do galaxies appear to recede from us the faster, the farther they are away? According to GR’s geometric interpretation, the answer in all four cases is: in virtue of the peculiarity of spacetime’s non-flat/non-Minkowskian geometry. In the Sun’s vicinity, the spacetime’s geometry deviates from spatial flatness: Mercury’s trajectory traces it; its orbit thus is a manifestation of this geometry. Likewise, in the case of the delayed radar echo: light traces out the spacetime geometry; in the presence of a mass this distortion elongates the path that light has to traverse; this results in a longer journey. Similar explanations essentially invoking the spacetime’s curved geometry as the germane explanans can be given for the other phenomena. Further examples from (astro-)physical practice abound: the Problem of Motion, i.e. the derivation of matter dynamics from GR’s constraints on spacetime (see e.g. Weatherall, 2017, 2019), explanations involving symmetries (for instance, in the context of conservation laws, see Lange, 2007, 2009), gravitational waves, gravitational collapse, energy extraction processes of black holes, etc. On the geometric interpretation of GR, spacetime structure *genuinely* and *ultimately* explains (some) aspects of the behaviour of matter.^34^

Are such geometric explanations non-causal, and in a suitable sense ‘metaphysical’? Do they qualify as instances of partial grounding? A fully satisfactory answer would require an account of causation as opposed to grounding; it would also require detailed case studies of paradigmatic examples. We don’t pretend to offer either. Three thoughts, however, suggest it (to our minds) as a prima facie plausible working hypothesis to conceive of the relation between matter and spacetime as (partial) grounding. We propose this as an idea worthwhile investigating in order to advance the debate.

First, eminent advocates (pace Brown, 2005, p. 24) of the geometric approach explicitly disavow that GR’s matter/spacetime relations (or spacetime-geometric explanations) are causal (e.g. Nerlich, 2007, Ch. 7).^35^ Three general problems indeed obstruct reference to causality in a general-relativistic context (Hoefer, 2009, Sect. 4.2). One is that approaches (such as Salmon’s and Dowe’s), based on conserved quantities, aren’t directly applicable to GR: the status of conservation of energy and momentum—as the two quantities standardly taken to be chiefly relevant—is controversial (Curiel, 2000; Dürr, 2020; Hoefer, 2000). Alternative accounts of causality, based on counterfactuals (such as Lewis) fare little better: in GR, one faces principled difficulties with regards to evaluating counterfactuals (Curiel, 2014; Jaramillo & Lam, 2019). GR poses a third problem for causality simpliciter: certain exotic spacetimes (see e.g. Hoefer, 2009; Sect. 4.1; Smeenk & Wüthrich, 2011), such as Gödel’s universe, enable causal loops or even time travel. Suppose that one is willing to take such spacetimes seriously as genuine possibilities. Then, such scenarios clash with received causal intuitions: accordingly, our hunches about causation must (at best) be radically revised. These revisions should be expected to affect the verdict whether the spacetime/matter inter-relation is causal or not.

Besides their being non-causal, spacetime-geometric explanations display two further salient features of a metaphysical nature. One is their greater modal scope than what is characteristic of causal explanations. Spacetime-geometric explanations abstract away from any details of matter (see e.g. Reutlinger & Saatsi, 2018; Saatsi, 2020). In fact, the form of necessity that spacetime structure affords seems to go beyond nomological/physical necessity: spacetime-geometric explanations even cover some counter-nomic scenarios; they explain certain phenomena also in worlds governed by laws different from ours. In this sense, recourse to spacetime discloses *deeper* law-like connections than the physical ones, familiar from, say, Maxwell’s Equations or the Dirac Equation.

Another distinctively metaphysical flavour of spacetime explanations is related. It stems from what one may call the *transcendental* function of spacetime structure in physical theorising: the formulation of laws of nature (usually implicitly) refers to spacetime structure (Curiel, 2016; Friedman, 1983; Norton, 2008); in order to write them down, one avails oneself of spatiotemporal posits.^36^ Spacetime theories, such as Special or General Relativity, explicate such implicit reference (ibid.; Sus, 2018).

In this sense, the dynamics of matter is adapted to spacetime structure (Maudlin, forth; Weatherall, forth.); spatiotemporal posits are built into their formulation. Thus construed, (classical) matter presupposes spacetime structure. Spatiotemporal posits encode necessary structural constraints that matter, subject to a particular dynamics must respect.^37^ Advocates of the geometric approach deny (e.g. op.cit.; Earman, 1989, Ch. 3) that one should understand this adaptation or necessity merely as a re-statement of the fact that the matter dynamics, has certain structural features; they deny that the claim that matter adverts to a certain spacetime structure is analytic (see e.g. Acuña, 2019; Myrvold, 2019): as realists about spacetime structure, its advocates view aspects of spacetime as explaining (some) behaviour of matter.

Spacetime’s transcendental function isn’t limited to the theoretical side of physics, i.e. the formulation of laws. Spacetime geometric aspects must also be *practically* presupposed in experimental contexts: typical empirical evidence for physical theories involves measurable quantities that possess time/length dimensions.

In short: according to our proposed metaphysical reading of the geometric approach to spacetime, spacetime structure partially grounds matter and its dynamical laws. With partial grounding a building relation in the sense of Bennett, spacetime (on Bennett’s account) or alternatively with the principle that *x*’s partial grounding of *y* implying *x*’s greater fundamentality, it follows that indeed spacetime is more fundamental than matter, as the super-substantivalist claims.

One may understand this turn of the argument as a generalisation of Lehmkuhl’s argument. The Energy–Stress Argument’s argumentative role is taken over by what above we called spacetime’s transcendental function: not only the (general-relativistic) energy–stress tensor, but *all* (classical) laws of nature presuppose spacetime structure. The Vacuum Argument’s purpose—to articulate a suitable sense in which spacetime can exist “independently” of matter—is reflected in spacetime’s *partial* grounding of matter structure: it only contributes to the latter; metaphysical worlds without matter are possible. Note that due to this generalisation, our account is extricated from the main problems with Lehmkuhl’s arguments, diagnosed in Sect. 3. Its scope extends to *all* of classical physics (classical particle mechanics and field theory). However, our proposal too requires the identification of the metric as spatiotemporal, i.e. **(INT-MET)**. We accept this as the price to pay for a weighty claim about spacetime’s metaphysical status: to draw metaphysical lessons from physics, interpretative decisions in particular are inevitable. That these aren’t indefeasible merely reflects that metaphysics, too, isn’t trafficking in infallible truths.

We’ll close with pre-empting two possible objections to our proposal. First, the Einstein Equations are often glossed as expressing “how matter tells spacetime how to curve”. Such a formulation accords matter the ability to influence or determine spacetime. If such influence or determination were cashed out in terms of partial ground, our proposal—with its tenet that spacetime partially grounds matter—would face an impasse: (partial) grounding is typically viewed as an *asymmetric* relation (see e.g. Fine, 2012); the partially grounded isn’t supposed to partially ground its partial ground. The objection can be warded off, however; the above characterisation of the Einstein Equation is specious.

The energy–stress tensor on the r.h.s. of the Einstein Equations *doesn’t* represent matter simpliciter (as Lehmkuhl, 2011 points out): at most it represents material energy–stress, a property of matter. As Lehmkuhl rightly emphasises, the energy–stress tensor itself depends on the metric. The Einstein Equations therefore give (formal/mathematical/functional) mutual *constraints* between the matter fields and the metric. As such they are neutral on any questions pertaining to what determines what: the Einstein Equations are merely implicit ways of singling out the physically appropriate (metric, matter fields) pairs. (Note that this neutrality doesn’t conflict with the claimed priority accorded to spacetime: that claim rested on independent arguments, such as spacetime’s transcendental function—over and above the Einstein Equations.)

The second complaint calling for a response is of a more metaphysical flavour: **(Exp-Dep-St)** states that what is partially grounded—and thus partially explained—is the *existence* of matter. On the other hand, the arguments we rehearsed, seem to explain *particular features* or (dynamical or kinematic) *behaviour* of matter in terms of spacetime structure. One might therefore conclude that spacetime falls short of partially grounding matter structure sensu stricto.

Two responses are possible. First, one could introduce a notion of dependence with regards to some respect *R* as follows:

*x* depends on *y* with respect to *R*: necessarily, there is something about *y*, namely its being *F*, that partially grounds the fact that *Rx*.

In the case at hand, the thought would be that matter depends on spacetime structure for (some of its) dynamic/kinematic properties:

Necessarily, there is something about spacetime, namely its structure – say, its curvature – that partially grounds, and thus partially explains, the fact that matter behaves in such and such a way.

Secondly, and relatedly, one can note that the relevant respect *R* is existence entailing. (That is: if *Rx* then *x* exists—more on this shortly.) If *R* is existence entailing, then the existence of *x* is partially grounded in *R*—one may argue. One can then invoke transitivity of partial grounding for the desired conclusion: spacetime structure partially grounds the existence of matter.

The first question to address then becomes: is *R* existence entailing? Berto (2012, p. 150) provides an exhaustive list of properties that may be taken not to be existence-entailing:

(L)ogical properties seem not to be existence entailing [...] The same seems to hold for counter-intentional properties – those having to do with being the object of some intentional state [...] If we like negative properties nonexistents can have plenty [...] Nonexistents can also currently possess the feature of having had certain properties in the past (e.g. having been blue-eyed). Analogously they may have modal properties, having to do with with the having of properties at other worlds.

It’ not difficult to see from the examples we give above that *R* doesn’t fit into any of these categories. Presumably *R* is the semantical value of a predicate, such as “moving in a certain way”. This seems to be existence entailing, if anything is. One wants to say that if something moves—in the sense of “being at different spatiotemporal locations”—it exists. This delivers what we are after: *R* is existence entailing, and *Rx* entails that *x* exists. As we pointed out already, one has to go a step further. That is, one has to endorse a second claim: that existing is partially grounded in exemplifying existence entailing properties. This, we admit, is controversial. We don’t want to defend the claim here. We merely claim that this is what a proponent of priority super-substantivalism needs to endorse for her argument to go through. Naturally one can turn the argument on its head.

Summing up: the best argument for priority super-substantivalism, we submit, is one that uses explanatory dependence to claim that matter explanatory depends (asymmetrically) on spacetime. From that, spacetime’s priority over matter follows. The crucial features behind the notion of explanatory dependence relate to modality, priority and fine-grainedness. First, explanatory dependence has the right kind of (i) modal *force* and (ii) modal *direction* to support super-substantivalist arguments. Second, explanatory dependence has in-built a notion of partial grounding that is usually taken to track relative fundamentality. Third, and finally, the notion of explanatory dependence is fine-grained. One can isolate *different* aspects of matter that are explained by—partially grounded in—*different* aspects of spacetime structure. This seems to chime with Lehmkuhl’s own conclusion: he who endorses this version of priority super-substantivalism.

can expect to learn something new about matter once he has associated it with particular aspects of spacetime structure, for the relationships between different aspects of spacetime structure we know of are likely to direct our attention to as yet unknown relationships between the different kinds of matter and their properties. The radical super-substantivalist may fail. But, if he succeeds, the reward is great (Lehmkuhl, 2018, p. 40).

To conclude we’d like to circle back to different versions of super-substantivalism we started with and provide a few details about their logical relations. Suppose (to our minds: plausibly) that, first, both constitution and “being more fundamental than’’ are asymmetric relations; and secondly, that constitution tracks relative fundamentality—that is, once again, it supports the conditional: if *x* (partly) constitutes *y*, then *x* is prior to *y*. Then, the following entailments go through:

(i) Identity Super-Substantivalism entails—(Priority Super-Substantivalism).
(ii) Identity Super-Substantivalism entails—(Constitution Super-Substantivalism);
(iii) By (i): Priority Super-Substantivalism entails—(identity Super-Substantivalism;
(iv) By (ii): Constitution Super-Substantivalism entails—(Identity Supersubstantivalism. (Thereby, the claim is vindicated that constitution isn’t identity.);
(v) Constitution Super-Substantivalism entails Priority Super-Substantivalism.

It is interesting to note that one *doesn’t get*:

(vi) Priority Super-Substantivalism entails Constitution Super-Substantivalism.

This non-entailment holds because claims of (relative) fundamentality/non-derivativeness, which form the core of Priority Super-Substantivalism, can be underwritten by relations very different from constitution. In this paper, we explored *explanatory dependence*. But there may be others.^38^

## Footnotes

1 Schaffer (2009) suggests a different classification. While he doesn’t consider what we label the Priority View, he also lists the **Eliminativist View**. According to the latter, there are no material objects. Lehmkuhl (2018, Sect. 6) proposes yet another classification, a partition into **radical** and **modest** super-substantivalism: the former seeks to reduce all physical properties to geometric ones, the latter—as a “purely” metaphysical position—“merely” allows spacetime to instantiate those physical properties directly that we normally attribute to matter.

2 Schaffer (2009, p. 140) equates constitution and identity. Consequently, the Constitution View and the Identity View collapse onto each other. Many constitution theorists would deny that constitution is identity. We follow suit: either constitution can be understood as a sui generis primitive relation, alternatively, one can define it, e.g. in mereological terms, as suggested by Gilmore (2014).

3 Throughout this paper, we’ll treat the two expressions as synonymous.

4 The subsequent construal of substance goes slightly against the letter of Lehmkuhl’s exposition. Lehmkuhl (2012, p. 27) seems to equate substances and fundamental entities simpliciter. Our explication of substance does remain faithful, though, to Lehmkuhl’s understanding: he explicitly presents the super-substantivalist’s spacetime as the bearer of properties (as does Schaffer).

5 Schaffer (2009) offers a beautiful simile (slightly streamlined). Let “pins” or “needles” stand for properties; and the “sewing table” for spacetime. Then, the spacetime’s substantiality means that the pins can be placed directly on the sewing table; one can dispense with any pin-cushions (placed on the table)—corresponding to matter as the usual carrier of properties—into which to stick them. Things are more complicated in other versions of super-substantivalism.

6 Albeit rarely explicated, this “rough” idea of ontological priority (i.e. priority_0_)_,_ seems to underly the common characterisations of substantivalism in terms of spacetime’s autonomous existence, an independence of the universe’s material content (cf. e.g. Maudlin, 1993, p. 202 or Norton, 2011, cited in Stachel, 2014). There are reasons to doubt that this *isn’t*, however, the most perspicuous way of characterising the debate. For example, North (2018) argues that the debate between relationalists and substantivalists should be construed as one about fundamentality—where that notion *doesn’t* necessarily coincide with independent/asymmetric existence, as asserted by priority_0_. We’ll revert to both issues in Sect. 4.

7 Lehmkuhl (2011, p. 471) points out that sometimes (e.g. in the case of massless electromagnetic fields) conformal structure suffices to define the energy–stress tensor. He points out, however, that the energy–stress tensor even in those (exceptional!) cases still display other forms of dependence. We’d like to add that conformal structure—pace Lehmkuhl (ibid.), who defines it as an equivalence class of metrics—is best understood as an irreducible *part* of a metric (see e.g. Stachel, 2011). (Together with the so-called volume part, it fully determines a metric.) As both the volume and the conformal part, on (INT-MET) (see below), receive a separate interpretation as spatiotemporal elements, even the definitional dependence on spacetime structure simpliciter goes through.

8 We’ll return to the issue of essential dependence in Sect. 4.

9 We thank an anonymous referee for stressing this.

10 The existence of vacuum solutions isn’t guaranteed in all theories. Arguably, for instance, Barbour-Bertotti theory (Barbour & Bertotti, 1977, 1982) doesn’t admit of (physical) vacuum solutions.

11 Note that, like GR, Newton-Cartan Theory has non-trivial so-called Weyl structure (see e.g. Dewar & Weatherall, 2018, Sect. 4, also Dewar & Read, ms).

12 The same holds for the “Jesus interpretation” of the metric, recently advocated by Martens and Lehmkuhl (2020a; 2020b): according to it, the metric has a matter-spacetime hybrid nature. As a result, “vacuum” solutions are *both* purely spatiotemporal and purely gravitational-material.

13 The non-dynamical nature of this plenum may look odd. But oddity isn’t tantamount to incoherence. Hence, the plenum interpretation remains a live option—at least at the (non-fundamental/effective) level of classical field theory. Furthermore, this oddity can be avoided: likewise relativistic field-theoretical proposals exist that explain the effects of a cosmological constant dynamically via a dynamical scalar field—so-called quintessence models (see e.g. Amendola & Tsujikawa, 2010, esp. Ch. 7). This scalar then plays the role of a plenum field.

14 Invoking such selection rules over and above a theory’s dynamical equations, in order to discriminate between physical and unphysical solutions needn’t be ad-hoc. It’s common practice in science to discard certain solutions as unphysical, for instance, due to considerations of instability, action-at-a-distance (in non-quantum theories), or causality violations.

15 Interestingly, both also contain physical singularities.

16 Note that already in the most conservative post-classical extension—the so-called semi-classical level (in which matter is described quantum theoretically, but on classical, general-relatistivistic spacetime background), technical difficulties occur for the energy–stress tensor (see e.g. Kiefer, 2012, Ch. 1.2; Maudlin et al., 2019).

17 Multi-metric theories of gravity are in fact an active area of research (e.g. Hohmann, 2014).

18 Note that it doesn’t take too much imagination to come up with non-Lagrangian theories: after all, once systems contain friction or other dissipative effects, a standard Lagrangian treatment is no longer viable. The reason stems from Hamilton’s principle, which the latter involves. However, it’s formulated for boundary conditions in time—not initial conditions (see e.g. Galley, 2014).

19 This account has a venerable philosophical pedigree. Arguably, its first appearance is in Aristotle (Met. 1019a1–4). We find it in Descartes’ *Principles of First Philosophy* and Spinoza’s *Ethics*. The contemporary *locus classicus* is Simons (1987, Ch. 7). Except for the anti-symmetry clause, Simons’ *Weak Foundation* (Simons 1987, p. 295) is exactly **(Ont-Prior)**.

20 We find such an understanding of substance in terms of independence in Aristotle, Descartes and Spinoza (see e.g. Robinson, nd). Lehmkuhl (2018, fn. 10) writes: "Schaffer (2009) traces back this [i.e. Lehmkuhl’s notion of substance, our emphasis] notion of substance to Aristotle, as well as Descartes and Spinoza". This could be taken to suggest that Lehmkuhl at least implicitly endorses the required independence of substances. It deserves to be pointed out that many pertinent authors in the philosophy of physics literature (e.g. Sklar, 1974 or Norton, 2019) indeed employ a notion of substance that involves “independent existence”.

21 By “relation *R* tracking relative fundamentality”, we simply mean that it supports the following conditional: if *aRb*, then either *b* is prior to *a*, or (depending on *R*) *a* is prior to *b*.

22 Therefore, appeal to *supervenience* is of no avail, either. We thank an anonymous reviewer for pressing us on this!

23 In order to claim the metaphysical advantages over the simple modal construal of ontological dependence, the injunction of essence has to be taken seriously. That is, one has to forgo reductionism about essences of the following type: **(RED-ESS)** ${\text{x}}$ is essentially ${\Phi }$, iff ${\text{x}}$ is necessarily ${\Phi }.$ Essentialists only retain the right to left direction of **(RED-ESS)**.

24 Note, however, that some self-identifying naturalists embrace an essentialist account of dependence [e.g. McKenzie (2014) and Calosi and Morganti (2020)]. McKenzie is explicit that commitment to essentialism isn’t as nefarious as it might appear from a naturalistic standpoint.

25 Vassallo (2019) argues that grounding is ill-suited to model dependence relations in GR. The main reason is that “(m)aterial facts depend on spatiotemporal ones and vice versa. Hence, the requirement of asymmetry for grounding is in stark tension with the (prima facie) mutual dependence encoded in the Einstein field equations” (Vassallo, 2019, p. 13). For reasons elaborated in Sect. 5, this argument doesn’t entirely sway us.

26 Note that Kane explicitly uses the word “hierarchy”. If one believes that “building” tracks relative fundamentality between levels, the passage seems to present a perfect example of a categorical account.

27 We don’t aim to stay faithful to the letter of Correia’s protrayal of categorical accounts.

28 In general, suppose one believes that wholes depend on their parts. Then consider any whole such that, both the whole in question and some of its parts belong to the same level. This gives a very general template for the holding of intra-level dependence relations.

29 The classical arguments wouldn’t be able to adjudicate between super-substantivalism and what Le Bihan (2018) calls “priority monism beyond spacetime”.

30 Perhaps the lesson to draw from the overall argument is that super-substantivalists should rest content to endorse a claim of relative fundamentality of spacetime over matter. Note that, strictly speaking, this entails that they would have to discard ST-FUND –see Sect. 2. Sincere thanks to an anonymous referee.

31 *x* is said to *partially* ground *y*, iff for some *y*, *x* together with some *x’* (“fully”) grounds *y*.

32 Here, we don’t employ the more standard ‘fact-talk’ when it comes to grounding. That is: one usually assumes that, if construed as a relation, the grounding relata belong to a specific ontological category, namely facts (for a classic reference see Fine, 2012). However, more liberal conceptions have been defended, permitting that the grounding relata can belong to any ontological category (e.g., events, objects, propositions, and so on), see e.g. Schaffer (2009).

33 Thanks to an anonymous referee for pushing us on this.

34 We don’t deny that there are *other* interpretations of GR besides the geometric one (recall Sect. 3)—interpretations in which geometry doesn’t enjoy pride of place as an explanans. Here, we needn’t embroil ourselves in the question which interpretation to go for. For our purposes, it suffices that that *a* prima facie plausible interpretation of GR—in fact, the received view—exists that strengthens Lehmkuhl’s argument.

35 We set aside metaphysically thin conceptions of causality (esp. regularity theories) or Einstein’s own (Ben-Menahem, 1993).

36 In Michael Friedman’s neo-Kantianism (2001, 2002) spacetime structure in fact serves as a form of apriori condition of the possibility of physical knowledge.

37 This isn’t to say that spacetime structure is *essential* for matter of a particular type: it seems counterintuitive to say that the essence of electromagnetic fields (if there is such a thing) hinges on whether they live on a flat or a curved spacetime; in both cases, the fields obey the same equations, and qualitatively have the same properties.

38 One such proposal we owe to an anonymous referee for this journal (sincere thanks!): **(EXP-DEP-ST-2)** Necessarily, the existence and behaviour of matter supervenes on the structure of spacetime; this supervenience itself is “a symptom” of an explanatory dependence of matter facts on facts about spacetime structure. A thorough assessment of **(EXP-DEP-ST-2)** deserves independent scrutiny. Here we simply note that it crucially features yet another notion of dependence – supervenience. One would need to address what, if anything, supervenience adds to the claim of explanatory dependence. Also, one would have to carefully address the logical relations between the two notions. These relations might turn out to be surprisingly complicated (see e.g., the papers in Hoeltje & Steinberg, 2013).
